# Zmienność Tolerancji Fenyloalaniny u Chorych Na PKU w Ciąży Pojedynczej i Bliźniaczej - Obserwacje i Doświadczenia z Jednego ośrodka Diagnostyczno-leczniczego (doniesienie Wstępne)

**DOI:** 10.34763/devperiodmed.20172104.344360

**Published:** 2018-01-02

**Authors:** Joanna Żółkowska, Kamil K. Hozyasz, Maria Nowacka

**Affiliations:** 1 Klinika Pediatrii, Instytut Matki i Dziecka, Warszawa, Polska; 2 Przykliniczna Poradnia Fenyloketonurii, Instytut Matki i Dziecka, Warszawa, Polska

**Keywords:** ciąża bliźniacza, cechy przebiegu ciąży, rozbieżność masy urodzeniowej, tolerancja fenyloalaniny, oddziaływania matczyno-płodowe, PKU, twin pregnancy, pregnancy characteristics, birthweight discordance, phenylalanine tolerance, maternal-fetal interactions, PKU

## Abstract

Podłożem fenyloketonurii (PKU) jest dziedziczony autosomalnie recesywnie niedobór hydroksylazy fenyloalaniny, który powoduje nadmierne gromadzenie się fenyloalaniny (Phe) we krwi i mózgowiu. Ograniczenie podaży Phe w diecie ma na celu umożliwienie utrzymywania się stężenia tego aminokwasu we krwi w rekomendowanym przedziale 120-360 μmol/L. W praktyce klinicznej ocena tolerancji Phe opiera się na częstych oznaczeniach stężenia aminokwasu w powiązaniu z analizą jadłospisów. Nieleczona PKU u kobiety w wieku prokreacyjnym może prowadzić do rozwoju zespołu fenyloketonurii matczynej u potomstwa.

**Celem pracy:**

była analiza wpływu ciąży wielopłodowej na tolerancję fenyloalaniny, wykorzystująca obserwacje ciąż pojedynczych i bliźniaczych u kobiet chorych na PKU stosujących dietę niskofenyloalaninową.

**Pacjenci i metody:**

Retrospektywnie analizowano ciążę pojedynczą i bliźniaczą u 3 chorych na PKU, stosujących dietę niskofenyloalaninową. Wszystkie pacjentki były objęte opieką wyspecjalizowanego dietetyka oceniającego tolerancję Phe. Analizowano wiek ciążowy wprowadzenia diety, przyrost masy ciała w czasie ciąży, odsetek oznaczeń Phe poza zakresem referencyjnym oraz pomiary noworodków, z których żaden nie chorował na PKU.

**Wyniki:**

Całkowity wzrost tolerancji Phe i jego wzorzec w ciążach pojedynczych i mnogich różnił się istotnie u każdej z pacjentek. W ciążach pojedynczych i mnogich tolerancja Phe wzrosła o 579%/468%, 674%/261% i 427%/236% u chorych z genotypem Q383X/R408W, EX3DEL/EX3DEL, R281L/R408W. W ostatnich 10 tygodniach ciąży wzrost tolerancji Phe sięgał odpowiednio 62%/149%, 33%/64% i 37%/40%. Stopień zwiększenia masy ciała ciężarnej oraz płodu nie umożliwiał przewidywania zmian tolerancji Phe.

**Wnioski:**

Poznanie tolerancji Phe w ciąży pojedynczej u chorej na PKU nie było pomocne w prognozowaniu dopuszczalnej podaży tego aminokwasu w ciąży bliźniaczej. Niezbędne są dalsze badania nad metabolizmem Phe w ciąży w celu opracowania zaleceń ułatwiających kompleksową opiekę nad chorymi na PKU w wieku rozrodczym.

## Abstract (en)

Phenylketonuria (PKU) is the autosomal recessive deficiency of phenylalanine hydroxylase resulting in the accumulation of phenylalanine (Phe) in blood and in the brain. Phe restriction in a patient’s diet is determined depending on the amount of Phe intake which allows for stable blood Phe levels within the therapeutic range of 120-360μmol/L. In clinical practice the empirical determination of Phe tolerance relies on frequent assessment of blood Phe concentrations in relation to Phe intake from food records. Untreated maternal PKU may lead to maternal PKU syndrome in offspring.

**The objective:**

of the study was to compare Phe tolerance during the course of singleton and multiple pregnancies of PKU patients.

**Case subjects and methods:**

The cases reviewed included three sets of classical PKU-affected Polish women on a low-phenylalanine diet during the course of singleton and twin pregnancies and their PKU-unaffected newborns. All the patients were under regular supervision of a metabolic dietitian to stabilize blood Phe levels and determine Phe tolerance. Data on pregnancy weight gain, the gestational age when the diet initiated, the percent of Phe assessments <120 μmol/L and >360 μmol/L, as well as offspring birth measurements were analyzed.

**Results:**

The total increase in Phe tolerance and its pattern during the course of singleton and twin pregnancies differed remarkably in each patient. Three PKU women (Q383X/R408W, EX3DEL/EX3DEL, R281L/R408W) increased their Phe tolerance in singleton and twin pregnancies by 579%/468%, 674%/261%, and 427%/236%, respectively. During the last 10 weeks of singleton and twin pregnancy Phe tolerance showed an increase by 62%/149%, 33%/64%, and 37%/40%, respectively. The analysis of predictors for Phe tolerance showed that an individual’s weight gain and the fetal weight gain as estimated from liveborn birth-weight data had no predictive capacity.

**Conclusions:**

Individual Phe tolerance in singleton pregnancies of PKU patients does not predict tolerance in twin pregnancy. Further research on the growing population of multiple pregnancy PKU patients is necessary to provide evidence-based guidelines to optimize the treatment of PKU in females of childbearing age.

Fenyloketonuria klasyczna (OMIM 261600; PKU) jest najczęściej występującym wrodzonym zaburzeniem metabolizmu aminokwasowo-białkowego [1]. Choroba dziedziczy się autosomalnie recesywnie i jest skutkiem mutacji genu *PAH*, powodujących zmniejszenie aktywności hydroksylazy fenyloalaninowej (PAH), która, głównie w wątrobie, przekształca aminokwas fenyloalaninę (Phe) w tyrozynę. Trwałemu uszkodzeniu ośrodkowego układu nerwowego i innym objawom PKU można skutecznie zapobiegać poprzez wczesne zastosowanie diety niskofenyloalaninowej [1, 2]. W 1956 r. Dent [3, 4] jako pierwszy wnioskował, a w 1963 r. Mabry i wsp. [4] przedstawili obserwacje potwierdzające zależność pomiędzy (nieleczoną) PKU u kobiety w ciąży a zaburzonym rozwojem jej potomstwa. W kolejnych latach udowodniono, że hiperfenyloalaninemia w ciąży może być przyczyną embriopatii, obejmującej m.in. zaburzenia wzrastania wewnątrzmacicznego, małogłowie, wady wrodzone, w tym wady serca, oraz nieprawidłowy rozwój psychiczny i intelektualny [5], co zbiorczo określa się terminem zespołu fenyloketonurii matczynej (MPKU). Na model zapobiegania MPKU składa się precyzyjne utrzymywanie ograniczonego dowozu Phe już od okresu prekoncepcyjnego, tak by: 1. stężenie we krwi tego aminokwasu mieściło się w arbitralnie ustalonym przedziale 120-360 μmol/L (2-6 mg%) a restrykcja nie kolidowała z ciążowym anabolizmem, 2. uzyskać pełne pokrycie zapotrzebowania na inne składniki pokarmowe i energię [2]. Nadal algorytmy stosowania diety niskofenyloalaninowej są stale niedoskonałe zwłaszcza w zakresie przewidywania zmian tolerancji Phe w przebiegu ciąży [2, 6].

Ciąże wielopłodowe, w praktyce w większości bliźniacze (97-98%), występują coraz częściej, co przede wszystkim wiąże się ze stosowaniem inwazyjnych technik wspomaganego rozrodu oraz wzrastającym wiekiem kobiet zachodzących w ciążę. W Europie na przełomie XIX i XX wieku częstość rodzenia się bliźniąt wynosiła 14-15/1000 porodów i zmalała do ok. 9-10/1000 porodów w latach 70-tych XX wieku, korelując z urbanizacją i obniżaniem się średniego wieku matek w coraz mniej dzietnych rodzinach. Ostatnie lata charakteryzuje szybki wzrost częstości ciąż bliźniaczych do ponad 15/1000 porodów (>3% noworodków stanowią bliźnięta). Ciąża mnoga stanowi czynnik ryzyka hipotro' i płodu i powikłań okołoporodowych [7, 8, 9].

Podobnie, jak w populacji ogólnej ciąże mnogie stanowią źródło problemów medycznych, a w części i etycznych, także w rodzinach osób chorych na PKU. Znane są przypadki selektywnej aborcji jednego bliźnięcia po ustaleniu rozpoznania PKU [10]. Badania preimplantacyjne, wykluczające PKU przeprowadzane na życzenie par o wysokim ryzyku posiadania chorego potomstwa, jak i inne procedury wspomaganego rozrodu, związane z transferem kilku zarodków, mogą prowadzić do ciąż wielopłodowych [10, 11]. Niestosowanie lub wadliwe prowadzenie diety niskofenyloalaninowej u chorej na PKU często skutkuje nieprawidłowym rozwojem płodu i decyzją o przerwaniu ciąży, zarówno pojedynczej, jak i mnogiej [12, 13, 14, 15, 16].

Badacze zajmujący się PKU wielokrotnie wykorzystywali model bliźniąt [17, 18, 19]. Wykazywano zmienność obrazu klinicznego choroby u osobników z tymi samymi mutacjami *PAH* porównując rodzeństwa, w tym zarówno bliźnięta dwujajowe [20], jak i jednojajowe [17, 21]. W 1995r. Verkerk i van der Meer z uniwersyteckiego szpitala w Maastricht [22] przedstawili, interesujący a niejasny co do pierwotnej przyczyny, przypadek noworodka płci męskiej z wysokim stężeniem Phe w 7 dniu życia i jego siostry bliźniaczki z prawidłowym wynikiem badania przesiewowego, u której do wczesnego rozpoznania PKU doszło dzięki bezwarunkowej dociekliwości klinicystów. W opisie 18 chorych na PKU z Rumunii 22% stanowiły bliźnięta jednojajowe [23]. Niekiedy jedno z bliźniąt, chore na PKU, swoimi potrzebami odnośnie terapii, przerasta wydolność rodziny i staje się przyczynkiem dla interwencji instytucji strzegących praw dziecka, która przypuszczalnie w przypadku niewystąpienia choroby nie byłaby konieczna [24].

Przedstawiano różny przebieg kliniczny ciąż i laktacji u jednojajowych bliźniaczek chorych na PKU [25, 26]. W 1979 r. Smith i wsp. [27] opisali ciężką wadę serca u dziecka matki chorej na PKU, urodzonej z ciąży dizygotycznej a leczonej dietą niskofenyloalaninową przez pierwszych 12 lat życia i po przerwie dopiero od 7 tygodnia ciąży. Pojedynczo raportowano przypadki MPKU w ciążach pojedynczych i bliźniaczej, zarówno mono- jak dizygotycznej, pacjentek nieleczonych [28,29]. W 1982r. Levy i wsp. [30] opisali kobietę, u której do rozpoznania PKU prowadziło stwierdzenie przejściowej hiperfenyloalaninemii u jednego z dwujajowych bliźniąt z małogłowiem w badaniu przesiewowym noworodków. Potomstwo z kolejnych ciąż bez włączania lub z relatywnie późnym włączeniem diety rozwijało się nieprawidłowo [30], co można uznać za typowy skutek braku leczenia. Obserwacje serii ciąż różnych pacjentek z PKU i ich potomstwa, wśród którego znajdowały się bliźnięta, przedstawiło kilka zespołów badaczy (1 ciąża bliźniacza spośród 5 opisanych [13], 1/30 [16], 1/39 [31], 2/48 [32] oraz 1/63 [14]). Naughten i Saul [32] opisali wystąpienie wady serca u obu bliźniąt urodzonych przez kobietę nie stosującą diety, jak i bliźniaczego potomstwa pacjentki będącej na diecie dopiero od 5 miesiąca ciąży. W publikacjach Maillot i wsp. [6] oraz Lee i wsp. [33] zawarto informacje o wykluczeniu ciąż bliźniaczych ze szczegółowych analiz zaburzonej prokreacji w PKU.

W 1984 r. Levy i wsp. [34] postulowali brak istotnego wpływu płodu na homeostazę Phe u ciężarnej chorej na PKU na podstawie analizy stężenia tego aminokwasu we krwi 4 matek z hiperfenyloalaninemią oraz w żylnej i tętniczej krwi pępowinowej podczas porodu, co było zgodne z wcześniejszymi obserwacjami porównywalnego ryzyka wystąpienia MPKU zarówno u potomstwa z jedną mutacją *PAH*, jak i z dwoma zmutowanymi allelami. U kobiet chorych na PKU, leczonych dietą niskofenyloalaninową, obserwuje się zazwyczaj istotny wzrost tolerancji Phe w ciąży [6]. W 2008r. Kohlschütter i wsp. [35] przedstawili interesujący opis pojedynczych ciąż u trzech kobiet chorych na PKU, stosujących dietę. U kobiet tych dwa płody były nosicielami jednej mutacji *PAH* a jeden płód był homozygotą *PAH* R408W. W ciążach z heterozygotycznym płodem udokumentowano systematyczny duży wzrost tolerancji Phe, natomiast ciążę z homozygotą mutacji *PAH* charakteryzowały trudności w dopasowaniu diety do zapotrzebowania białkowo-kalorycznego, co skutkowało m.in. dużym przyrostem masy ciała ciężarnej. Próby zwiększenia podaży newralgicznego aminokwasu bezwarunkowo skutkowały przekroczeniami górnej granicy dopuszczalnego stężenia Phe we krwi [35]. Na tej podstawie wnioskowano o istotnym (klinicznie) uczestnictwie wątroby płodu w metabolizmie Phe u leczonych ciężarnych z PKU.

Celem pracy była analiza wpływu ciąży wielopłodowej na tolerancję fenyloalaniny, wykorzystująca obserwacje ciąż pojedynczych i bliźniaczych u kobiet chorych na PKU stosujących dietę niskofenyloalaninową.

## Uczestniczki badania i metody

Przeprowadzono retrospektywną analizę dokumentacji medycznej kobiet chorych na PKU z wywiadem ciąży mnogiej i pojedynczej, podczas których leczono je dietą niskofenyloalaninową w Poradni Fenyloketonurii przy Klinice Pediatrii IMiD. Zidenty' kowano 3 pacjentki, posiadające potomstwo z jednej ciąży bliźniaczej i jednej pojedynczej. W momencie zajścia w ciążę wiek kobiet mieścił się w przedziale 24-32 lata.

Analizowano tolerancję Phew okresie prekoncepcyjnym oraz w ciąży w zależności od liczby płodów, masy ciała i jej przyrostu u ciężarnej, wskaźnika masy ciała (BMI) ciężarnej, masy urodzeniowej potomstwa i estymowanych jej zmian w okresie życia płodowego z wykorzystaniem siatek centylowych dla dzieci z ciąż pojedynczych i bliźniaczych [36, 37]. Zmiany tolerancji Phe obliczano na podstawie dostarczanych przez pacjentki dzienników diety w wytypowanych okresach. Stopień rozbieżności masy urodzeniowej bliźniąt obliczano według wzoru:

$$\frac{\text{ masa większego - masa mniejszego }}{\text{ masa większego }}\times 100\text{\%}.$$

Zgodnie z piśmiennictwem rozbieżność co najmniej 15% uznano za istotną klinicznie [38, 39].

## Wyniki

U wszystkich trzech chorych na PKU pierwsza ciąża była pojedyncza (tab. I). Średni wiek zajścia w ciążę pojedynczą i bliźniaczą wynosił odpowiednio: 25 ±2 lata i 29 ±2 lata. Wskaźnik masy ciała (BMI) przed zajściem w ciąże bliźniaczą był większy niż przed ciążą pojedynczą u dwóch z trzech pacjentek (tab. I). Różnica pomiędzy końcowym BMI w ciąży bliźniaczej i pojedynczej mieściła się w zakresie 0,1-5,4. Tylko u jednej chorej w każdej z dwóch ciąż dietę niskofenyloalaninową stosowano już od okresu prekoncepcyjnego (tab. I). U wszystkich trzech kobiet odsetek nieprawidłowych wysokich wyników oznaczeń stężenia Phe we krwi był większy w ciążach bliźniaczych, natomiast odsetek nieprawidłowo małych stężeń Phe był większy w ciążach jednopłodowych u dwóch ciężarnych (ryc. 1, 2 i 3; tab. I). U każdej ciężarnej dobowa podaż białka i energii była większa w ciąży bliźniaczej w porównaniu z pojedynczą (tab. I).

**Tabela I:** Charakterystyka 6 ciąż, w tym 3 bliźniaczych, u trzech pacjentek chorych na fenyloketonurię. Table I. Clinical data on 6 pregnancies in three PKU patients resulting in live births, including 3 sets of twins.

		Pacjentka 1/Patient 1 *Genotyp/Genotype* (Q383X/R408W)		Pacjentka 2/Patient 2 *Genotyp/Genotype* (EX3DEL/EX3DEL)		Pacjentka 3/Patient 3 *Genotyp/Genotype* (R281L/R408W)	
		Ciąża pojedyńcza *Singleton pregnancy*	Ciąża bliźniacza *Twin pregnancy*	Ciąża pojedyńcza *Singleton pregnancy*	Ciąża bliźniacza *Twin pregnancy*	Ciąża pojedyńcza *Singleton pregnancy*	Ciąża bliźniacza *Twin pregnancy*
Wiek pacjentki w momencie zajścia w ciążę *Patient's age at conception* [lata/yrs]		28	32	24	28	23	28
Procedura wspomagania In vitro *in vitro* fertilization [Y/N]		N	N	N	N	N	N
Palenie w ciąży *Smoking during pregnancy [Y/N]*		N	N	N	N	N	N
Masa ciała przed ciążą *Prepregnancy weight* [kg]		41,1	38,8	61	72	62,0	77,8
BMI przed ciążą *Prepregnancy BMI* [kg/m^2^]		17,12	16,2	21,8	25,7	22,15	27,8
Przybytek masy ciała ciąży *Pregnancy weight gain* [kg]		13,9	16,3	15	6	21	20,2
BMI przed porodem *BMI before delivery* [kg/m^2^]		22,9	23	27	26,1	29,64	35
Przybytek masy ciała w pierwszym ciąży *Weight gain in first trimester* [kg]		1,6	3,1	0	-1	-0,8	1,2
BMI w pierwszym trymestrze ciąży *BMI in first trimester*		17,8	17,46	21,8	25,36	21,86	28,2
Stosunek masy urodzeniowej potomstwa do przybytku masy ciała ciężarnej *Newborn(s) weight as a proportion of maternal weight gain*		0,24	0,26	0,24	1,02	0,15	0,32
Stężenia phe przed zajściem w ciążę *Preconceptional phe level* [μmol/L]		516	309	1763	740	260	915
Wiek ciąży podczas wprowadzenia diety niskofenyloalaninowej *Gestational age when diet initiated [tyg./weeks]*		0	0	16	1	0	4
% wyników oznaczeń stężenia Phe>360μmol/L w całej ciąży *% of Phe assessments>360μmol/L during whole pregnancy*		8	12	11	31	17	25
% wyników oznaczeń stężenia Phe<120μmol/L w całej ciąży *% Phe assessments<120μmol/L during whole pregnancy*		19	45	30	20	30	15,6
Preparat leczniczy *Dietary formula*		XP Maxamum	XP Maxamum	XP Maxamum	PKU cooler Milupa PKU3 tern pora	PKU Lophlex LQ3	Phenyl-free 2HP Milupa PKU3 advanta PKU Lophlex LQ
Dobowa podaż białka z preparatu *Daily protein intake from dietary formula* [g]^1^	14 Hbd	58,5 (1,3)	48,75 (1,2)	b.d.	88 (1,3)	70 (1,1)	98,82 (1,3)
	28 Hbd	58,5 (1,23)	78 (1,5)	97,5 (1,4)	108 (1,6)	90 (1,3)	102 (1,2)
	34 Hbd	78 (1,4)	87,75 (1,6)	97,5 (1,3)	115 (1,5)	100 (1,2)	109,32 (1,2)
Dobowa podaż energii *Daily energy intake* [kcal]	14 Hbd	1638- 1911	1725 - 2204	b.d.	2435 - 3017	1056-1178	2300-2500
	28 Hbd	1645 - 2238	1654 - 2428	2301-2501	1637-2563	1692-2250	2500-2800
	34 Hbd	1589 - 2489	1500 - 2300	2048 - 2614	2114-3076	2079-2282	2500-2800

1 W nawiasie g/kg masy ciała//n *parenthesis g/kg of body weight;* b.d. - brak danych/no *data*

Ciąże mnogie zakończyły się wcześniej od pojedynczych o średnio 2,7 tygodnia (tab. II). Bliźnięta były dwujajowe tej samej płci różnej od płci rodzeństwa z ciąży pojedynczej. Najmniejsza urodzeniowa masa ciała potomstwa z ciąży pojedynczej i mnogiej wynosiła odpowiednio 3120 g i 1890 g. Różnica masy ciała w parach potomstwa sięgała od 40 g do 680 g. W dwóch ciążach wystąpił istotny klinicznie stopień rozbieżności urodzeniowych mas ciała (pacjentka 1-17%, pacjentka 3-19%). Najmniejsze obwody głowy stwierdzono u bliźniąt (30 cm; <10c) pacjentki 1, urodzonych w 35 tygodniu ciąży, i donoszonego noworodka (31 cm; -3 SD) z pojedynczej ciąży chorej, która dietę niskofenyloalaninową wprowadziła dopiero w drugim trymestrze ciąży (tab.II). U żadnego z dzieci nie rozpoznano PKU a stężenia Phe w przesiewowym badaniu noworodków nie przekraczały 113 μmol/L (1,89 mg%). Poza jednym przypadkiem spodziectwa, nie stwierdzono u potomstwa innych wad budowy ciała o podłożu zaburzonej organogenezy (tab. II).

**Tabela II:** Zestawienie pomiarów urodzeniowych potomstwa trzech chorych na fenyloketonurię. Table II. Birth measurements for completed pregnancies of three PKU patients.

		Tydzień ciąży *Weeks gestation*	Płeć Sex	Masa ciała *Weight [g; percentyl]*	Długość ciała *Length [cm]*	Obwód głowy *Head circumference [cm; percentyl]*	Punktacja Apgar *Apgar score*	Wady wrodzone, cechy dysmorfii *Congenital anomalies, dysmorphic features [Y/N]*	Phew przesiewie noworodków *Phe concentration^1^ [μmol/L]*	Status chorego *PKU-affection [Y/N]*
Pacjentka 1 *Patient 1*	Ciąża poj. *Singleton*	38	Ż	3280/50^2^	55	33/ >10^2^	10	N	113	N
	Bliźnięta dwujajowe *Fraternal twins*	35	M	2290<25-50^3^	46	30/< 10c^4^	8/9	N	74	N
		35	M	1890< 10-25 ^3^	46	30/< 10c^4^	4/7/7/7	N	65	N
Pacjentka 2 *Patient 2*	Ciąża poj. *Singleton*	40	M	3600/75^2^	50	31/<3^2^	9	N	113	N
	Bliźnięta dwujajowe *Fraternal twins*	38	Ż	3090/50-75^3^	53	33/ 25-50^4^	9/10/10	N	80	N
		38	Ż	3030/50-75 ^3^	54	34/ 50 -75 ^4^	10	N	71	N
Pacjentka 3 *Patient 3*	Ciąża poj. Singleton	40	Ż	3120<50^2^	55	35/ >75^2^	9/10	N	81	N
	Bliźnięta dwujajowe *Fraternal twins*	37	M	3580>97^3^	57	37/ >90^4^	10	N	60	N
		37	M	2900/50-75^3^	53	34/ 50 -75^4^	10	spodziectwo hypospadias	52	N

1 Whole blood phenylalanine concentration at newborn screening ^2^ Według standardów WHO/*According to WHO standards* [36] ^3^ Według ogólnopolskich siatek centylowych dla bliźniąt. Projekt *DOM/According to Dom project – Polish percentile charts for twins* [40] ^4^ Według angielskich siatek centylowych dla *bliźniąt/According to head circumference standards for English twins* [41]

Zwiększenie dobowej tolerancji Phe przez całą ciążę wynosiło od 506 mg (ciąża mnoga, pacjentka 2) do maksymalnie 1338 mg (ciąża pojedyncza, pacjentka 1), ryc. 1, 2 i 3. Zaawansowaniu ciąży towarzyszył wzrost tolerancji Phe w stosunku do masy ciała chorych na PKU (tab. III). U pacjentki 1 i 3 w ciąży bliźniaczej tolerancja Phe osiągała podobne wartości co w ciąży pojedynczej w tym samym wieku (tab. III i IV). U pacjentki 2 tolerancja Phe była skrajnie, nawet ponad dwukrotnie (w 29 tygodniu ciąży), mniejsza w ciąży bliźniaczej, osiągając przed rozwiązaniem nieco ponad 60% wartości wyliczanej dla ciąży pojedynczej (ryc. 2 i tab. IV). Pomiędzy 25 a 34 tygodniem ciąży tolerancja Phe w przeliczeniu na szacowaną masę płodu/ów zmniejszyła się w ciąży pojedynczej i mnogiej u pacjentki 1, 2 i 3 odpowiednio o: 49,8% i 34%, 52,2% i 45,9% oraz 32,6% i 46,8% (tab. IV). W tym samym okresie tolerancja Phe w przeliczeniu na szacowaną masę płodów w ciążach bliźniaczych stanowiła od 46% do 63%, od 23% do 30% i od 29% do 49% wartości z ciąż pojedynczych odpowiednio u pacjentki 1, 2 i 3 (tab. IV).

**Tabela III:** Szacunkowa tolerancja fenyloalaniny i jej stosunek do masy ciała ciężarnej w ciążach pojedynczych i bliźniaczych. Table III. Estimated phenylalanine (Phe) tolerance and its ratio to weight of pregnant woman in singleton and twin pregnancies.

Wiek ciąży *Week gestation* [tygodnie]	Pacjentka 1 *Patient 1*						Pacjentka 2 *Patient 2*					
	Ciąża pojedyncza *Singleton pregnancy*			Ciąża bliźniacza *Twin pregnancy*			Ciąża pojedyncza *Singleton pregnancy*			Ciąża bliźniacza *Twin pregnancy*		
	Phe stężenie level^1^ [μmol/L]	Phe tolerancja^2^ [mg]	Phe/weight^3^ [mg/kg]	Phe stężenie level^1^ μmol/L	Phe tolerancja^2^ [mg]	Phe/weight^3^ [mg/kg]	Phe stężenie level^1^ [μmol/L]	Phe tolerancja^2^ [mg]	Phe/weight^3^ [mg/kg]	Phe stężenie level^1^ μmol/L	Phe tolerancja^2^ [mg]	Phe/weight^3^ [mg/kg]
14	100 (40-157)	408 (321-495)	9,6 (7,5-11,6)	173 (103;244)	449 (400-499)	2,11 (9,75-12,2)	867	-	-	109 (307-129)	196,5 (194-199)	2,77 (2,73-2.8)
28	264	1011,5 (963- 1060)	20,23 (19,3-21,2))	118	937,5 (756-1119)	1 O 1 (14,6-21,6)	415	954 (950-958)	14 (13,9-14)	209 (88-330)	420,5 (400-441)	5,76 (5,48-6,04)
Przedostatni *Pre-week delivery*	367	(13931480 -1569)	(25,226,8 -28,4)	228	(12501275 -1300)	(22,723,1 -23,6)	501	(11021121 -1140)	(14,514,75 -15)	156	700	8,97
**Wiek ciąży *Weeks gestation* [tygodnie]**	Pacjentka 3 *Patient3*											
	**Ciąża pojedyncza** *Singleton pregnancy*			**Ciąża bliźniacza** *Twin pregnancy*								
	**Phe stężenie level^1^ [μmol/L]**	**Phe tolerancja^2^** [mg]	**Phe/weight^3^ [mg/kg]**	**Phe stężenie level^1^** [μmol/L]	**Phe tolerancja^2^** [mg]	**Phe/weight^3^ [mg/kg]**						
14	138	334,5 (321-348)	5,46 (5,24-5,69)	168	260	3,3						
28	68 (59-77)	559 (437-681)	7,76 (6,06-9,45)	161	655 (650-660)	7,9 (7,83-7,95)						
Przedostatni *Pre-delivery week *	292 (283-301)	1035 (938-1132)	12,47 (11,3-13,63)	387	b.d	b.d.						

1 średnie z co najmniej dwóch oznaczeń/average *of minimum two assessments;* ^2^uśredniona rekonstrukcja co najmniej dwóch jadłospisów/average *of minimum two diet reconstructions;* ’stosunek tolerowanego spożycia Phe do aktualnej masy ciała ciężarnej/*Ratio of tolerated amount of Phe to weight of pregnant woman* b.d. brak danych/no *data*

**Tabela IV:** Szacunkowa tolerancja fenyloalaniny w ciążach pojedynczych i bliźniaczych z uwzględnieniem masy ciała płodów. Table IV. Estimated phenylalanine (Phe) tolerance and its ratio to fetal weight in singleton and twin pregnancies.

Wiek ciąży *Weeks gestation* [tygodnie]	Pacjentka 1 *Patient 1*						Pacjentka 2 *Patient 2*						Pacjentka 3 *Patient 3*					
	Ciąża pojedyncza *Singleton pregnancy*			Ciąża bliźniacza *Twin pregnancy*			Ciąża pojedyncza *Singleton pregnancy*			Ciąża bliźniacza *Twin pregnancy*			Ciąża pojedyncza *Singleton pregnancy*			Ciąża bliźniacza *Twin pregnancy*		
	Phe stężenie level^1^ [μmol/L]	Phe tolerancja^2^ [mg]	Phe/fetal weight^3^ [mg/kg]	Phe stężenie level^1^ [μmol/L]	Phe tolerancja^2^ [mg]	Phe/fetal weight^3^ [mg/kg]	Phe stężenie level^1^ [μmol/L]	Phe tolerancja^2^ [mg]	Phe/fetal weight^3^ [mg/kg]	Phe stężenie level^1^ [μmol/L]	Phe tolerancja^2^ [mg]	Phe/fetal weight^3^ [mg/kg]	Phe stężenie level^1^ [μmol/L]	Phe tolerancja^2^ [mg]	Phe/fetal weight^3^ [mg/kg]	Phestężenie level^1^ [μmol/L]	Phe tolerancja^2^ [mg]	Phe/fetal weight^3^ [mg/kg]
25	159 (126-191)	862 (734-990)	1098 (935-1261)	209	662 (523-801)	509 (402-616)	114 (88;145)	654 (600-708)	833 (764-902)	265	347 (342-352)	222 (219-226)	175 (53-179)	555,5 (470-641)	707,6 (537,1-816,5)	104	460	263
29	290	1023 (968-1078)	742 (702-781)	306 (225-386)	1091 (957-1225)	464 (407-521)	438	946,5 (934-959)	686 (677-695)	202 (156;248)	436 (427-445)	161 (158-165)	144 (104-184)	682 (540-824)	494,6 (391,6-579,5)	b.d.	b.d.	b.d.
30	142 (138-164)	984,5 (783-1186)	631 (502-760)	196	1009 (940-1078)	396 (369-423)	186	901 (845-957)	578 (542-614)	292 (338;245)	462 (451-473)	149 (145-153)	112 (58-166)	770,5 (677-864)	494,2 (434,2-554,2)	178	818 (706-930)	220 (190-250)
31	112 (102-121)	1112 (861-1363)	640 (492-788)	66 (31-101)	1007 (857-1156)	353 (301-406)	152	854,5 (850-859)	488 (485-491)	228 (74;377)	471,5 (460-483)	135 (131-138)	133	797 (768-826)	455,2 (438,6-471,7)	b.d.	b.d.	b.d.
32	166 (174-158)	1171 (1059-1283)	600 (542-657)	199 (137-260)	1146 (1068-1224)	360 (336-385)	160	852 (847-857)	436 (434-439)	127 (177;77)	466,5 (458-475)	123 (121-125)	161 (136-185)	868 (683-1053)	444,4 (349,7-539,2)	497	925 (900-950)	218 (212-224)
33	188	1411,5 (1310-1513)	653 (606-700)	260	1275 (1250-1300)	364,3 (357-374)	239	871 (850-893)	403 (393-413)	82 (74;91)	520,5 (480-561)	121 (112-131)	98	854,5 (802-907)	395,2 (232,2-419,5)	182	801 (712-890)	170 (151-189)
34	302	1311 (1056-1566)	551,4 (444-658,8)	228	1275 (1250-1300)	332 (326-339)	243	945,5 (934-957)	398 (393-403)	168 (105;232)	550,5 (546-555)	120 (119-121)	104	1134,5 (1069-1200)	477,3 (449,7-504,8)	326	720 (600-840)	140 (117-163)
35	194	1770 (1650-1890)	682 (636-728)	499	b.d.	b.d.	245	947,5 (946-959)	367	117 (35;199)	613,5 (549-678)	123 (110-136)	469 (459-479)	1247,5 (1078-1417)	480,7 (415,4-546,1)	484	b.d.	b.d.
36	173 (157-190)	1327 (1066-1588)	472 (377-565)	b.d.	b.d.	b.d.	122	954,5 (949-960)	339 (337-341)	166 (127;203)	638,5 (614-663)	119 (114-123)	332 (247-416)	993,5 (870-1117)	353,2 (309-397,1)	387	b.d.	b.d.
37	347	1304 (1154-1455)	431 (381-481)	b.d.	b.d.	b.d.	168	1029 (948-1110)	340 (313-366,5)	160 (169; 150)	673 (646-700)	117 (112-122)	475	b.d.	b.d.	153	b.d.	b.d.
38	301	1481 (1393-1569)	489 (460-518)	b.d.	b.d.	b.d.	145	1105 (1096-1113)	341 (339-344)	156	700	114	264 (263-266)	1113,5 (935-1292)	344,1 (289-399,3)			
39							501	1121 (1102-1140)	326 (321-332)	b.d.	b.d.		283 (264-301)	1035 (938- 1132)	301,3 (273-329,5)			

1 średnie z co najmniej dwóch *ozmczen/average of minimum two assessments;*^2^ uśredniona rekonstrukcja co najmniej dwóch jadłospisów/average *of minimum two diet reconstructions;*^3^ *Stosunek* tolerowanego spożycia Phe do estymowanej masy płodu(ów)/ *Ratio of tolerated amount of Phe to estimated weight of fetus(ses)* b.d. - brak danych / *no data*

U każdej chorej stwierdzono większy wzrost tolerancji Phe w ciąży pojedynczej niż bliźniaczej zarówno przez pierwsze 34 tygodnie (cezura przyjęta w związku z zakończeniem się ciąży bliźniaczej pacjentki 1 w 35 tygodniu i dostępnością ostatnich obliczeń tolerancji Phe z przedostatniego tygodnia) jak i przez cały czas jej trwania (tab. V). Jednakże w ostatnich 10 tygodniach ciąży mniejszy wzrost tolerancji Phe w ciąży pojedynczej w porównaniu do ciąży bliźniaczej, bezwzględny i procentowy, stwierdzono u pacjentki 1 (tab. V).

**Tabela V:** Wzrost tolerancji fenyloalaniny w różnych okresach ciąży. Table V. The increase in phenylalanine tolerance during the course of pregnancy.

		Szacunkowy wzrost tolerancji fenyloalaniny *Estimated increase in phenylalanine tolerance^1^*									
		Pierwszy trymestr *First trimester*		Drugi trymestr *Second trimester*		Pierwsze 34 tygodnie ciąży *During the first 34 weeks *		Ostatnie 10 tygodni ciąży *During thelast 10 weeks*		Przez całą ciążę *During the whole pregnancy*	
		mg	%	mg	%	mg	%	mg	%	mg	%
Pacjentka 1 *Patient 1*	Ciąża pojedyncza *Singleton pregnancy*	149(231-380)	64,5	651 (321-972)	202	1335(231-1566)	578	601(968-1569)	62	1338(231-1569)	579
	Ciąża bliźniacza *Twin pregnancy*	243(229-472)	106	464(400-864)	116	1069(231-1300)	463	777(523-1300)	149	1071(229-1300)	468
Pacjentka 2 *Patient 2*	Ciąża pojedyncza *Singleton pregnancy*	b.d.	b.d.	(146663-809)	454	(146811-957)	555	(850280-1130)	33	(146984-1130)	674
	Ciąża bliźniacza *Twin pregnancy*	-42(247-205)	-17	178(194-372)	92	308(247-555)	125	273(427-700)	64	506(194-700)	261
Pacjentka 3 *Patient 3*	Ciąża pojedyncza *Singleton pregnancy*	177(200-377)	88,5	378 (321-699)	117	1000(200-1200)	500	287(768-1055)	37	855(200-1055)	427
	Ciąża bliźniacza *Twin pregnancy*	29(250-279)	11,6	267 (260-527)	103	590(250-840)	236	240(600-840)	40	590(250-840)	236

1 W oparciu o rekonstruowane przeciętne jadłospisy/*Based on average diet reconstructions* W nawiasie podano zarejestrowaną wartość najmniejszą i największą/*In parentheses the recorded min and max values*b.d. brak danych/no *data*

## Dyskusja

W 2009r Didycz i wsp. [42] opisali 50 ciąż u 21 pacjentek z hiperfenyloalaninemią, objętych specjalistyczną opieką w Małopolsce. Wywiad położniczy jednej kobiety obejmował pierwszą ciążę bez stosowania diety niskofenyloalaninowej, z której hipotroficzne dziecko zmarło, oraz nieplanowaną kolejną ciążę z dietą od 6 tygodnia jej trwania, zakończoną w 40 tygodniu narodzinami zdrowych bliźniąt o masie ciała 2130 g i 2240 g.

Nasza praca stanowi pierwszą w Polsce, oraz, w świetle znanego nam piśmiennictwa, pod względem szczegółowości opracowania przypuszczalnie także pionierską na świecie, próbę analizy serii ciąż mnogich u kobiet chorych na PKU na diecie niskofenyloalaninowej (włączonej najpóźniej od 4 tygodnia ciąży).

Wielkość wzrostu Phe przez całą ciążę pojedynczą a także jej zmiany w okresie bezpośrednio poprzedzającym rozwiązanie, czy ich powiązanie z masą ciała ciężarnej oraz estymowaną masą płodu, nie umożliwiały przewidywania ilościowej tolerancji Phe w ciąży bliźniaczej. Postulowanego, przez badaczy niemieckich, proporcjonalnego udziału wątroby płodu, bez defektu metabolizmu, w zwiększaniu tolerancji Phe [35] nie potwierdziły nasze obserwacje ciąż bliźniaczych. Dalszych badań będzie wymagać wyjaśnienie, dlaczego nawet w ciąży z dwoma dużymi płodami, o łącznej masie urodzeniowej ponad dwukrotnie większej niż w ciąży pojedynczej (pacjentka 3), całkowita tolerancja Phe była mniejsza a po przeliczeniu na masę płodową pomiędzy 30 i 34 tygodniem ciąży nie osiągała nawet 50% wartości stwierdzanej w ciąży pojedynczej. Uwzględniając dynamikę wzrastania tolerancji Phe w ostatnich tygodniach ciąży bliźniaczej, jedynie w przypadku niewystąpienia porodu przedwczesnego (35 tydzień ciąży) u pacjentki 1 można byłoby oczekiwać zrównania lub przekroczenia wartości z ciąży pojedynczej. Niemniej w przeliczeniu na masę płodową do 34 tygodnia ciąży tolerancja w ciąży bliźniaczej była o ponad połowę mniejsza niż w ciąży pojedynczej.

Mniejszy w porównaniu z ciążą pojedynczą wzrost tolerancji Phe w ciąży bliźniaczej u kobiet chorych na PKU jest zaskakujący. Dla wyjaśnienia tego zjawiska niezbędne będzie odstąpienie od praktyki wyłączania pacjentek z ciążą mnogą z prac obserwacyjnych poświęconych zmianom tolerancji Phe u chorych w wieku prokreacyjnym [6,33], co przypuszczalnie umożliwi zgromadzenie danych dla wnioskowania o ' zjopatologicznym podłożu. Ciąża wielopłodowa jest powiązana ze zwiększoną aktywnością mało poznanych mediatorów płodowo-łożyskowych, oddziałujących na metabolizm kobiety [43, 44, 45, 46], co skutkuje m.in. pobudzeniem wydzielania hormonów tarczycowych [47], które część białek transportowych, jak LAT1 i LAT2, wspólnie wykorzystują z dużymi neutralnymi aminokwasami (jak np. Phe) [48, 49], oraz syntezy niektórych białek w wątrobie [50]. Na obecnym etapie poznania zagadnienia można tylko wysunąć wstępną hipotezę, że w porównaniu do ciąży pojedynczej zwiększonemu zapotrzebowaniu na Phe i jej przemianie w wątrobie płodów w ciąży bliźniaczej może towarzyszyć zmieniona dystrybucja fenyloalaniny i przyspieszony metabolizm białkowy w organizmie matki chorej na PKU, przyczyniający się do zwiększenia puli wolnej Phe.

**Ryc. 1:**
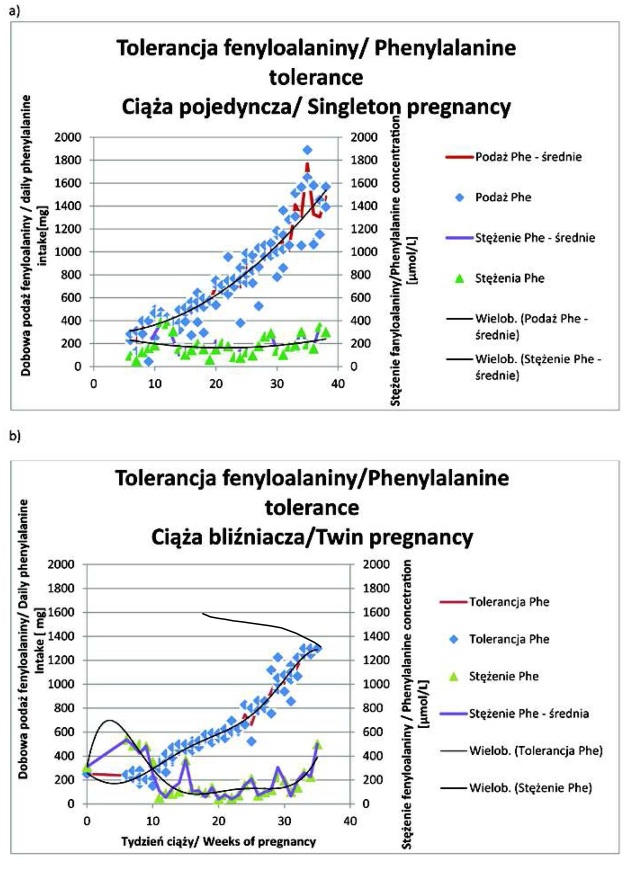
Tolerancja fenyloalaniny u pacjentki 1 w ciąży pojedynczej (a ) i bliźniaczej (b). Fig. 1. Phenylalanine tolerance in singleton (a) and twin (b) pregnancy of patient 1.

**Ryc. 2:**
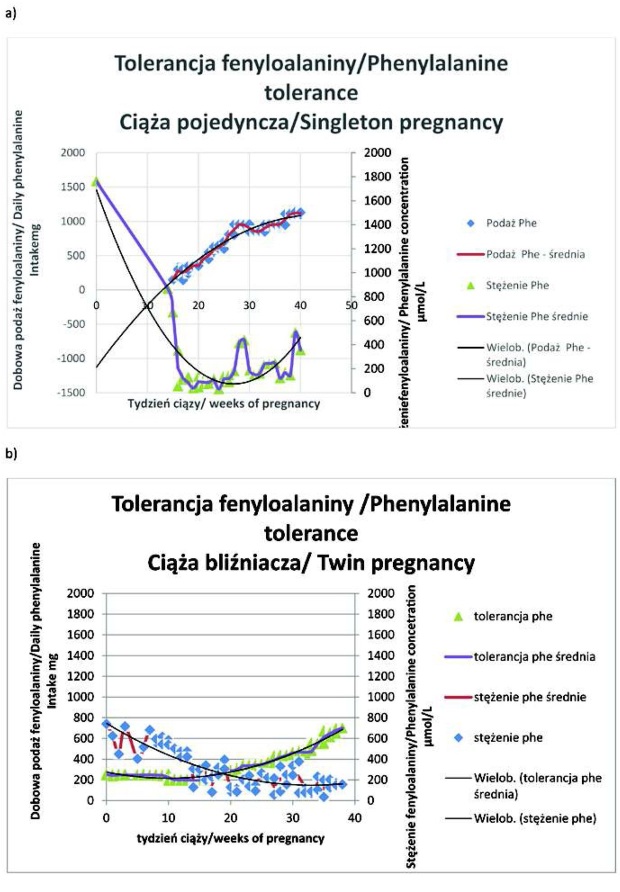
Tolerancja fenyloalaniny u pacjentki 2 w ciąży pojedynczej (a ) i bliźniaczej (b). Fig. 2. Phenylalanine tolerance in singleton (a) and twin (b) pregnancy of patient 2.

**Ryc. 3:**
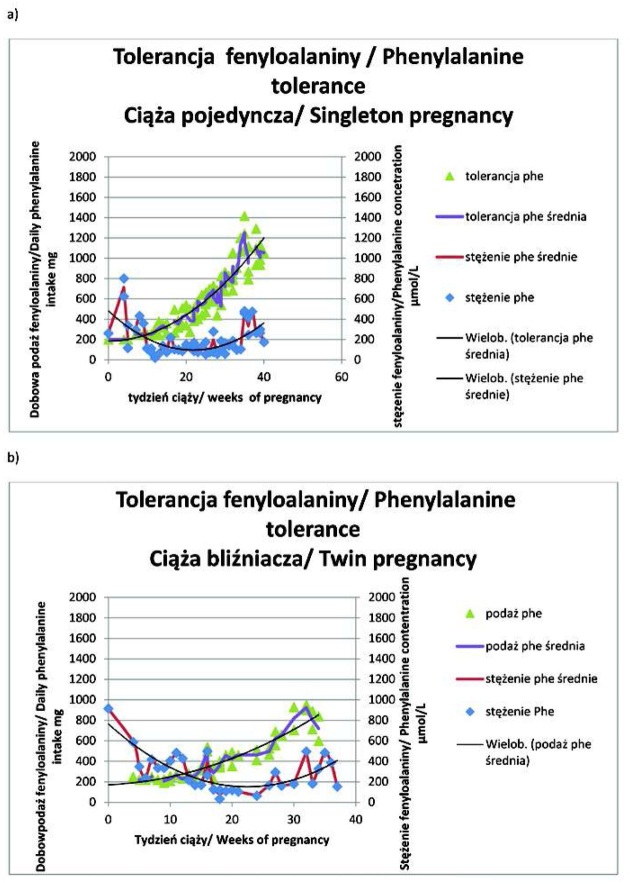
Tolerancja fenyloalaniny u pacjentki 3 w ciąży pojedynczej (a ) i bliźniaczej (b). Fig. 3. Phenylalanine tolerance in singleton (a) and twin (b) pregnancy of patient 3.

W 1977 r. na podstawie przeglądu piśmiennictwa Pueschel i wsp. [51] sugerowali częste występowanie PKU u potomstwa kobiet z tą chorobą (17/197 /8,6%/ dzieci 61 pacjentek). Obecnie przyjmuje się, że w populacjach pochodzenia europejskiego u potomstwa kobiet chorych na PKU ryzyko odziedziczenia zmutowanego genu *PAH* od ojca, będącego bezobjawowym nosicielem, wynosi tylko ok. 1:120 [12]. U żadnego z dziewięciorga dzieci, urodzonych z przedstawianych przez nas ciąż, nie rozpoznano PKU ani nie występowała przejściowa hiperfenyloalaninemia. Opisywano przypadki spodziectwa u chorych na PKU [52] i ich potomstwa [53], jednakże wada ta nie uchodzi za objaw MPKU. Spodziectwo częściej występuje u dzieci z ciąż mnogich i z wywiadem wspomaganej implantacji zarodka [54, 55]. W naszym materiale spodziectwo stwierdzono u bliźnięcia z ciąży nieplanowanej, ale w której dietę niskofenyloalaninową stosowano już od 4 tygodnia ciąży, czyli przed okresem krytycznym rozwoju embrionalnego cewki moczowej [56]. Urodzeniowy obwód głowy tego noworodka przekraczał 50c. Relatywnie najmniejszy obwód głowy stwierdziliśmy u syna pacjentki 2 z pierwszej ciąży pojedynczej, w której dopiero w 16 tygodniu rozpoczęto ścisłe stosowanie diety niskofenyloalaninowej. Pomimo prawidłowego rozwoju intelektualnego (IQ 90) i niewystępowania zaburzeń neurologicznych, utrzymujące się izolowane małogłowie (obwód głowy w 4 r.ż. − 47,5 cm, -3 SD) uprawnia do wskazywania MPKU jako jego pierwotnej przyczyny [2, 12, 16, 53].

Istotną niedoskonałością naszej pracy było objęcie analizą ciąż, w których diety niskofenyloalaninowej nie wprowadzono prekoncepcyjnie, co stanowi aktualnie ogólnie przyjętą rekomendację [2, 6]. Populacja polska charakteryzuje się wysoką częstością ciąż nieplanowanych a realizując przytoczoną rekomendację należałoby ściśle nadzorować leczenie dietetyczne u wszystkich kobiet chorych na PKU w wieku rozrodczym, w tym nieodczuwających korzyści dla własnego zdrowia z uciążliwej na co dzień terapii. Porównania pomiędzy pacjentkami byłyby obiektywniejsze, gdyby u nich resztkowa aktywność hydroksylazy fenyloalaninowej miała te same podłoże molekularne w zakresie mutacji genu *PAH*. U dwóch z 3 pacjentek występowała ponad 15% procentowa różnica masy ciała przed ciążą pojedynczą i bliźniaczą a przypuszczalnie oddziaływanie na płód przyrostu masy ciała kobiety w ciąży zależy od jej przedciążowego BMI [57, 58]. Poza tym przeliczenia tolerancji Phe na estymowaną masę płodów dizygotycznych, szczególnie przy występujących różnicach ich wielkości i braku danych o popłodzie, obciąża duże uproszczenie [39, 43].

Jako zalety pracy wymagają podkreślenia homogenność etniczna pacjentek, niestosowanie technik wspomaganego rozrodu oraz niewystępowanie ekspozycji na dym tytoniowy, jak i nadzorowanie diety niskofenyloalaninowej przez tego samego dietetyka we wszystkich ciążach.

## Wnioski

Poznanie tolerancji Phe w ciąży pojedynczej u chorej na PKU i jej zależności od wzrostu masy ciała matki i płodu nie umożliwia satysfakcjonującego prognozowania dopuszczalnej podaży tego aminokwasu w ciąży bliźniaczej. Opracowanie zaleceń ułatwiających kompleksową opiekę nad chorymi na PKU w wieku rozrodczym wymaga dalszych badań.

### Podziękowania

*Wszystkie oznaczenia stężenia fenyloalaniny wykonano w Zakładzie Badań Przesiewowych i Diagnostyki Metabolicznej Instytutu Matki i Dziecka, Kierownik: dr Mariusz Ołtarzewski. W dotarciu do archiwalnych pozycji piśmiennictwa nieocenionej pomocy udzielili bibliotekarze mgr Ewa Plewko (Biblioteka Naukowa Instytutu Matki i Dziecka) i mgr Grzegorz Święćkowski (Biblioteka Naukowa Narodowego Instytutu Zdrowia Publicznego – Państwowego Zakładu Higieny)*.
